# Post-marketing safety of immunomodulatory drugs in multiple myeloma: A pharmacovigilance investigation based on the FDA adverse event reporting system

**DOI:** 10.3389/fphar.2022.989032

**Published:** 2022-12-01

**Authors:** Tingting Jiang, Hui Su, Yanping Li, Yuanlin Wu, Yue Ming, Chen Li, Ruoqiu Fu, Lu Feng, Ziwei Li, Li Li, Rui Ni, Yao Liu

**Affiliations:** ^1^ Department of Pharmacy, Daping Hospital, Army Medical University, Chongqing, China; ^2^ State Key Laboratory of Biotherapy and Cancer Center, West China Hospital, and Collaborative Innovation Center of Biotherapy, Sichuan University, Chengdu, China

**Keywords:** IMiDs, multiple myeloma, FAERS, adverse event, pharmacovigilance, data mining

## Abstract

**Objective:** In recent years, the emergence of immunomodulatory drugs (IMiDs) has significantly improved clinical outcomes in patients with multiple myeloma (MM); however, serious adverse events (AEs) have hindered their safe clinical application. This study aimed to characterize the safety profiles and differences in IMiDs through a disproportionality analysis using the U.S. Food and Drug Administration Adverse Event Reporting System (FAERS), a post-marketing surveillance database.

**Methods:** This study filtered reports of thalidomide, lenalidomide, and pomalidomide as primary suspect drugs in FAERS files from January 2013 to December 2021. AEs in the reports were retrieved according to the preferred terms (PTs) of the Medical Dictionary for Regulatory Activities. Furthermore, we detected safety signals using the reporting odds ratio (ROR), proportional reporting ratio (PRR), and Bayesian belief propagation neural network (BCPNN). When all three algorithms showed an association between the target drug and the AE, a positive signal was generated.

**Results:** We extracted 9,968 thalidomide, 231,926 lenalidomide, and 55,066 pomalidomide AE reports. AEs were more common in male patients and in those >44 years old. Important safety signals were detected based on the system organ classes (SOC), including thalidomide (cardiac disorders: ROR, 2.87; PRR, 2.79; IC 1.22), lenalidomide (gastrointestinal disorders: ROR, 2.38; PRR, 2.27; IC 0.75), and pomalidomide (respiratory, thoracic, and mediastinal disorders: ROR, 2.14; PRR, 2.09; IC 0.85). Within the PT level, we identified novel risk signals: the thalidomide-induced second primary malignancy (SPM) signal was significant; lenalidomide reduced the success rate of hematopoietic stem cell collection; and three IMiDs may cause human chorionic gonadotropin increase, but this needs to be proven by clinical data. Pneumonia, sepsis, and renal failure are common risk factors for death due to IMiDs. Compared with thalidomide and lenalidomide, pomalidomide has a lower risk of venous thromboembolism (VTE) and is beneficial to patients with renal insufficiency.

**Conclusion:** Mining data from FAERS resulted in novel AE signals, including adenocarcinoma of colon, harvest failure of blood stem cells, and increased levels of human chorionic gonadotropin. Further investigation is required to verify the significance of these signals. Moreover, IMiDs showed differences in safety reports, which should be emphasized by clinicians.

## 1 Introduction

Multiple myeloma (MM) is one of the most common hematological malignancies, accounting for 20% of deaths from hematopoietic cancers and nearly 2% of cancer-related deaths (San Miguel, 2015; Naymagon and Abdul-Hay, 2016; Sonneveld and Broijl, 2016). Clinically, MM is characterized by malignant proliferation of plasma cells in the bone marrow, and monoclonal immunoglobulins in blood or urine, causing anemia, renal insufficiency, extensive bone destruction, hypercalcemia, and repeated severe infections (Fernández-Lázaro, 2020). Currently, MM is incurable (Hemminki et al., 2021). Traditional standard induction therapy for MM includes corticosteroids, melphalan, prednisone, or a combination of vinblastine, doxorubicin, and dexamethasone. However, due to increased resistance and drug-related adverse events (AEs) associated with classical chemotherapy and glucocorticoids, the median overall survival (OS) of MM patients is still not optimistic. Recently, the prognosis of MM patients has dramatically improved with the emergence of immunomodulatory drugs (IMiDs) and proteasome inhibitors (PIs) as evidenced by the increase in complete remission (CR) rates from 5% to 30% and extension of OS from 3 years to 5–15 years (Rajkumar, 2013; Kyle and Rajkumar, 2014).

Currently, three IMiDs have been approved to treat MM: thalidomide, lenalidomide, and pomalidomide (Palumbo et al., 2008; Scott and Lyseng-Williamson, 2011; Elkinson and McCormack, 2013). IMiDs exert anticancer effects through various mechanisms such as inducing tumor cell apoptosis, disturbing the interaction of tumor cells with stromal marrow cells, and increasing antitumor immune responses (Fernández-Lázaro et al., 2018; Charlinski et al., 2021). IMiDs exhibit moderate cross-reactivity and permissible sequential therapy; therefore, they can be applied to treat all stages of MM (Raza et al., 2017). Meanwhile, IMiDs are also the standard of care for patients who are suitable or unsuitable for induction therapy of autologous stem cell transplantation (ASCT), as maintenance therapy after ASCT, and receive relapsed/refractory MM (RRMM) treatment (Charlinski et al., 2021). Multiagent combinations based on IMiDs can prolong progression-free survival and OS and improve the quality of life (Miguel et al., 2013; Jones et al., 2016a; Garderet et al., 2018; Richardson et al., 2019; Siegel et al., 2020; Charlinski et al., 2021). Due to durable objective response rates, pomalidomide has been recommended as first-line and second-line treatment for lenalidomide-resistant and bortezomib-sensitive patients, respectively, according to the EHA-ESMO guidelines (Dimopoulos et al., 2021). However, further clinical practice and research revealed that IMiDs may cause serious AEs, such as rash, constipation, and venous thromboembolism (VTE) (Lonial et al., 2011; Ocio et al., 2012). Surprisingly, although the chemical structures of IMiDs are similar, their AEs were different. During thalidomide treatment, teratogenicity, sedation, and peripheral neuropathy were observed. Ito et al. identified cereblon as the primary target of thalidomide teratogenicity (Terpos et al., 2015; Holstein and McCarthy, 2017). The incidence of VTE significantly increased when thalidomide and lenalidomide were combined with conventional chemotherapy drugs (Musallam et al., 2009). Studies have demonstrated that patients receiving lenalidomide have an increased risk of second primary malignancies (SPMs), especially hematological malignancies (Razavi et al., 2013). Pomalidomide-associated fatal AEs have also been reported, including pneumonia, cardiac arrest, and progressive multifocal leukoencephalopathy (PML) (Richardson et al., 2019; Health Canada, 2022). Unfortunately, related research that directly compares the safety of the three IMiDs is scarce. Additionally, differences in the safety of IMiDs may affect treatment decisions and medication adherence.

Surveillance of post-marketing adverse drug events is critical for clinically rational drug use, with most IMiD-related AEs coming from clinical trials. However, clinical trials are usually limited by scale and ethics, and it is difficult to conduct large-scale preventive clinical studies to comprehensively analyze all types of patients (Beaulieu-Jones et al., 2020; Roberts and Ferguson, 2021). Therefore, real-world data are needed to supplement or verify clinical trials and to understand the safety profile of IMiDs better. Large real-world databases of AEs are the main data source for safety assessment of marketed drugs with fast-tracking and priority review, such as the U.S. Food and Drug Administration Adverse Event Reporting System (FAERS), the largest publicly available pharmacovigilance database (Health Canada, 2022). It contains patient data outside clinical trials and can be used for post-marketing surveillance (Raschi et al., 2019; Raschi et al., 2020).

To provide an overview of the safety profiles of IMiDs, we retrospectively analyzed real-world AEs of IMiDs from the first quarter of 2013 (2013Q1) to the fourth quarter of 2021 (2021Q4) by mining data from FAERS.

## 2 Materials and methods

### 2.1 Data collection and source

We downloaded all reports from 2013Q1 to 2021Q4 from the publicly available FAERS database (FDA, 2022). Each quarterly report contains seven datasets: patient demographics (DEMO), drug (DRUG), reaction (REAC), outcome (OUTC), report source, therapy, and indications for use; the DEMO, DRUG, REAC, and OUTC datasets were used in this study and are linked by the primary ID that identifies FAERS reports. Following FDA recommendations, we deduplicated the data in two steps: first, by filtering unique row variables; second, by selecting the latest case version with the same CASEID and removing redundant records. Reports for the following terms representing IMiDs were qualified: “Thalomid”, “Thalidomide”, “Distaval”, “Contergan”, “Revlimid”, “Lenalidomide”, “Pomalidomide”, and “Pomalyst”. Only reports with the drug code “prime suspect” were collected for analysis.

### 2.2 Definition of adverse events

AEs in the FAERS database were coded according to the preferred terms (PTs) of the Medical Dictionary for Regulatory Activities (MedDRA version 25.0) (Medical Dictionary for Regulatory Activities, 2022). MedDRA is multiaxial in that a PT can be linked to multiple system organ classes (SOCs), but each PT is assigned a single primary SOC. The extracted AEs can be associated with the corresponding SOCs through the hierarchical structure of MedDRA. In this study, we only analyzed the primary SOC associated with PT to avoid repetitive counting. Any significant AE not listed on the label was defined as an unexpected adverse drug reaction. To minimize the risk of indication bias (whereby the drug indication is reported as an AE), we removed PTs associated with the drug indication and complications in MM for analysis (Huang et al., 2020); i.e., we only analyzed drug-induced AEs and not disease states.

### 2.3 Data mining and analysis

We detected AE signals using three algorithms: the reporting odds ratio (ROR), proportional reporting ratio (PRR), and Bayesian confidence propagation neural network (BCPNN) (Ahmed et al., 2009; Poluzzi et al., 2009; Sakaeda et al., 2013). These methods are based on a two-by-two contingency (Supplementary Table S1) and can be used to investigate the statistical association between a drug and AE to detect potential AE signals. To avoid false-positive signals, the criterion is achieved only when all three algorithms show that the frequency and signal intensity between a drug and AE. Subsequently, it is determined as disproportionality, prompting the generation of a positive signal (Supplementary Table S2) (van Puijenbroek et al., 2002). Microsoft EXCEL 2019 and SPSS 26.0 statistical software were used for data analysis.

## 3 Results

### 3.1 Descriptive analysis

During the 9-year study period from January 2013 to December 2021, FAERS received a total of 11,209,429 AE reports, with 9,968 for thalidomide (0.09%), 231,926 for lenalidomide (2.07%), and 55,066 for pomalidomide (0.49%). The characteristics of the IMiD AE reports are described in Table 1. Male patients had a slight advantage compared with female patients, and there was a higher proportion of patients aged >44 years. The majority of reports were from the United States, Japan, and Canada and were submitted by physicians, pharmacists, and other health professionals, which accounted for the highest percentage of reports for thalidomide (31.61%), while pharmacists accounted for the highest percentage of reports for lenalidomide (40.23%) and pomalidomide (44.31%).

**TABLE 1:** Characteristics related to immunomodulatory drugs (IMiDs) safety reports from January 2013 to December 2021.

	Thalidomide	Lenalidomide	Pomalidomide
	N^a^ (%)	N^a^ (%)	N^a^ (%)
Number of adverse events reports	9968 (100)	231926 (100)	55066 (100)
Sex			
Female	4457 (44.71)	111893 (48.25)	26326 (47.81)
Male	4942 (49.58)	116132 (50.07)	28072 (50.98)
Unknown	569 (5.71)	3901 (1.68)	668 (1.21)
Age (year)			
<18	187 (1.88)	131 (0.06)	28 (0.05)
18–44	317 (3.18)	1919 (0.83)	349 (0.63)
45–64	1737 (17.43)	34322 (14.80)	8205 (14.90)
65–74	1724 (17.30)	41986 (18.10)	10540 (19.14)
≥75	1489 (14.94)	43427 (18.72)	8830 (16.04)
Unknown	4514 (45.28)	110141 (47.49)	27114 (49.24)
Reporters			
Consumer	1145 (11.49)	12213 (5.27)	2552 (4.63)
Physician	2877 (28.86)	47425 (20.45)	11014 (20.00)
Other health-professional	3151 (31.61)	58993 (25.44)	12049 (21.88)
Pharmacist	2178 (21.85)	93312 (40.23)	24401 (44.31)
others	447 (4.48)	18949 (8.17)	4768 (8.66)
Unknown	170 (1.71)	1034 (0.45)	282 (0.51)
Reporter country			
United States	8491 (85.18)	212858 (91.78)	50821 (92.29)
Japan	10 (0.10)	4368 (1.88)	1548 (2.81)
Canada	68 (0.68)	2115 (0.91)	722 (1.31)
Others	1399 (14.03)	12585 (5.43)	1975 (3.59)

^a^ Number of patients with adverse events.

### 3.2 Outcomes and fatality of IMiDs-related AEs

**FIGURE 1:**
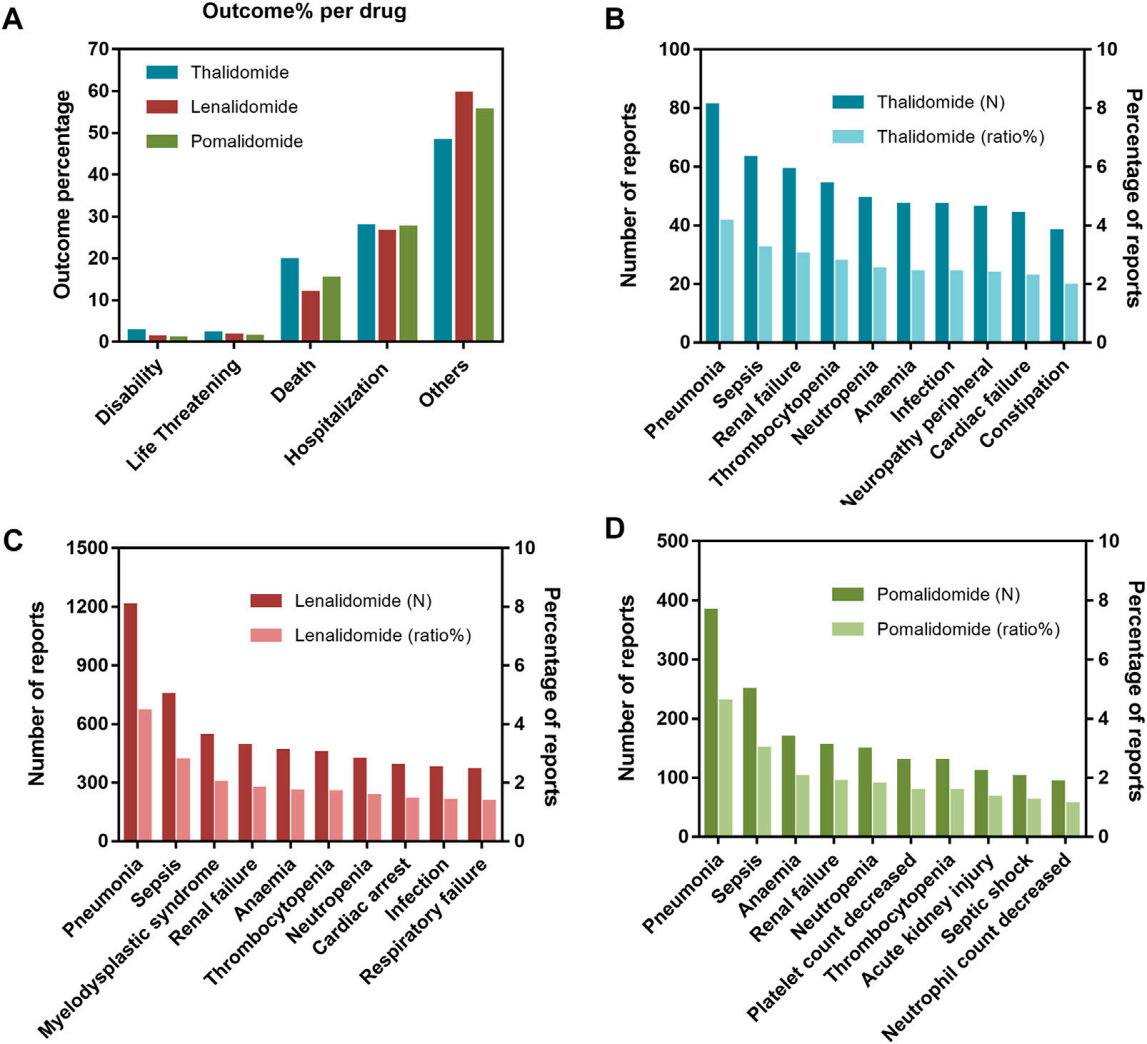
**(A)** Outcomes for adverse events (AEs) associated with immunomodulatory drugs (IMiDs). **(B)** The top 10 AEs leading to death for thalidomide. **(C)** The top 10 AEs leading to death for lenalidomide. **(D)** The top 10 AEs leading to death for pomalidomide.

Nearly 50% of AE reports described serious outcomes (Figure 1A), with a higher proportion of hospitalizations (initial or prolonged) and deaths. A peak in the reporting of hospitalization (initial or prolonged) and death was noted for thalidomide (27.70% and 19.65%, respectively), while lenalidomide had the lowest percentage among the drugs studied (26.30% and 11.72%, respectively). To further investigate the AEs leading to death, we separately evaluated the mortality (according to the number of deaths reports) caused by different AEs among the three drugs. Among them, deaths due to pneumonia and sepsis were ranked as the top two reasons for thalidomide (Figure 1B), lenalidomide (Figure 1C), and pomalidomide (Figure 1D). By analyzing the population characteristics, we found that death due to pneumonia and sepsis was more common in middle-aged and elderly male patients, especially those >65 years of age (Table 2).

**TABLE 2:** Clinical characteristics of deaths due to immunomodulatory drugs (IMiDs) related pneumonia and sepsis in the FAERS database (January 2013 to December 2021).

Pneumonia		Sepsis	
Sex	N^a^ (%)	Sex	N^a^ (%)
Female	556 (33.27)	Female	392 (36.91)
Male	946 (56.61)	Male	589 (55.46)
Unknown	169 (10.11)	Unknown	81 (7.63)
Age (year)		Age (year)	
<18	4 (0.24)	<18	4 (0.38)
18–44	7 (0.42)	18–44	9 (0.85)
45–64	249 (14.90)	45–64	211 (19.87)
≥65	1038 (62.12)	≥65	597 (56.21)
Unknown	373 (22.32)	Unknown	241 (22.69)

^a^ Number of patients with adverse events.

### 3.3 Disproportionality analysis

#### 3.3.1 Analysis of AEs at the SOC level

These AEs were classified according to the corresponding SOC of MedDRA involving 27 SOCs. The most frequently reported SOCs for thalidomide and lenalidomide were “infections and infestations,” “neoplasms benign, malignant and unspecified (incl cysts and polyps)”, and “investigations,” while for pomalidomide, the most commonly reported were “infections and infestations,” “investigations,” and “gastrointestinal disorders” (Supplementary Table S3). Within the SOCs, we conducted disproportionate analysis to assess the association between AEs and organs; the larger the ROR, PRR, and IC values, the stronger the correlation (van Puijenbroek et al., 2002). There are certain differences in the SOC involved with the IMiDs, as shown in Table 3: there were four significant safety signals for thalidomide (cardiac disorders, skin and subcutaneous tissue disorders, metabolism and nutrition disorders, and vascular disorders); AE reports of lenalidomide focused on gastrointestinal disorders and musculoskeletal and connective tissue disorders; and pomalidomide correlated with four SOCs (metabolism and nutrition disorders, respiratory, thoracic and mediastinal disorders, skin and subcutaneous tissue disorders, and musculoskeletal and connective tissue disorders).

**TABLE 3:** Detected significant safety signals based on system organ class (SOC).

SOC	N^a^ (%)	ROR (95% CI)	PRR (χ^2^)	IC (IC-2SD)
Thalidomide				
Cardiac disorders	86 (4.13)	2.87 (3.67–2.24)	2.79 (76.69)	1.22 (0.43)
Skin and subcutaneous tissue disorders	115 (5.53)	2.56 (3.16–2.07)	2.47 (81.34)	1.09 (0.41)
Metabolism and nutrition disorders	57 (2.74)	2.25 (3.02–1.68)	2.21 (30.92)	0.95 (0.01)
Vascular disorders	63 (3.03)	2.18 (2.88–1.65)	2.14 (31.53)	0.92 (0.02)
Lenalidomide				
Gastrointestinal disorders	434 (7.65)	2.38 (2.71–2.08)	2.27 (174.84)	0.75 (0.37)
Musculoskeletal and connective tissue disorders	216 (3.81)	2.08 (2.49–1.74)	2.04 (66.61)	0.66 (0.13)
Pomalidomide				
Metabolism and nutrition disorders	82 (2.79)	2.45 (3.18–1.89)	2.41 (49.30)	0.99 (0.17)
Respiratory, thoracic and mediastinal disorders	145 (4.94)	2.14 (2.60–1.77)	2.09 (62.97)	0.85 (0.23)
Skin and subcutaneoustissue disorders	133 (4.53)	2.07 (2.53–1.69	2.02 (52.84)	0.81 (0.17)
Musculoskeletal and connective tissue disorders	124 (4.22)	2.06 (2.53–1.67)	2.01 (48.78)	0.81 (0.15)

^a^ Number of patients with adverse events.

#### 3.3.2 Analysis of AEs at the PT level

According to the criteria of the three algorithms, we identified 81, 292, and 189 suspicious signals for thalidomide, lenalidomide, and pomalidomide, respectively. Supplementary Table S4 presents a list of the 20 most frequently reported AEs. We found that the most frequently reported AEs for thalidomide were peripheral neuropathy (*n* = 544), pneumonia (*n* = 362), and unevaluable events (*n* = 306); for lenalidomide, there was a higher percentage of Diarrhea (*n* = 15527), fatigue (*n* = 13794), and pneumonia (*n* = 10916); for pomalidomide, pneumonia (*n* = 3,683), fatigue (*n* = 3,299), and decreased white blood cell count (*n* = 1,980) accounted for a relatively high proportion. Among the AEs, nasopharyngitis caused by pomalidomide was not included in the label. The top 20 PTs associated with statistical significance for IMiDs are shown in Table 4. The number of AEs not listed on the label were nine for thalidomide: human chorionic gonadotropin increased, medulloblastoma, myelofibrosis, squamous cell carcinoma of the skin, rectal adenocarcinoma, adenocarcinoma of the colon, malignant brain neoplasm, basal cell carcinoma, non-small cell lung cancer; two for lenalidomide: human chorionic gonadotropin increased; blood stem cell harvest failure; and one for pomalidomide: human chorionic gonadotropin increased.

**TABLE 4:** Top 20 preferred terms (PT) associated with immunomodulatory drugs (IMiDs) for signal strength.

PT	N^a^ (%)	ROR (95% CI)	PRR (χ2)	IC (IC-2SD)
Thalidomide	21045 (100)			
Human chorionic gonadotropin increased	34 (0.16)	126.18 (88.92–179.05)	125.98 (3894.97)	4.76 (3.62)
Neuropathy peripheral	544 (2.58)	17.19 (15.78–18.72)	16.77 (7991.69)	4.01 (3.73)
Full blood count decreased	131 (0.62)	17.41 (14.64–20.69)	17.30 (1990.61)	3.93 (3.36)
Medulloblastoma	14 (0.07)	564.16 (305.64–1041.34)	563.79 (5747.54)	3.86 (1.95)
Myelofibrosis	25 (0.12)	22.78 (15.35–33.83)	22.76 (512.47)	3.62 (2.33)
Light chain analysis increased	16 (0.08)	41.74 (25.40–68.60)	41.71 (618.90)	3.61 (2.01)
Squamous cell carcinoma of skin	35 (0.17)	16.57 (11.87–23.12)	16.54 (505.64)	3.52 (2.43)
Unevaluable event	306 (1.45)	10.52 (9.39–11.78)	10.38 (2580.15)	3.32 (2.95)
Rectal adenocarcinoma	11 (0.05)	44.67 (24.52–81.37)	44.65 (456.09)	3.26 (1.35)
Adverse drug reaction	288 (1.37)	9.65 (8.58–10.84)	9.53 (2187.81)	3.20 (2.82)
Acute myeloid leukaemia	52 (0.25)	10.79 (8.21–14.18)	10.76 (457.42)	3.18 (2.28)
Adenocarcinoma of colon	15 (0.07)	18.33 (11.02–30.51)	18.32 (242.71)	3.13 (1.49)
Peripheral sensory neuropathy	24 (0.11)	12.61 (8.44–18.85)	12.60 (254.22)	3.10 (1.79)
Brain neoplasm malignant	22 (0.10)	11.91 (7.83–18.12)	11.90 (217.98)	3.01 (1.64)
Deep vein thrombosis	171 (0.81)	7.72 (6.64–8.98)	7.67 (987.52)	2.88 (2.38)
Myelodysplastic syndrome	40 (0.19)	8.45 (6.19–11.53)	8.43 (260.66)	2.83 (1.81)
Full blood count increased	9 (0.04)	19.85 (10.28–38.32)	19.85 (159.00)	2.78 (0.70)
Basal cell carcinoma	44 (0.21)	7.78 (5.79–10.47)	7.77 (258.33)	2.75 (1.78)
No therapeutic response	34 (0.16)	7.48 (5.34–10.48)	7.47 (189.58)	2.65 (1.55)
Non-small cell lung cancer	10 (0.05)	8.86 (4.76–16.50)	8.86 (69.29)	2.36 (0.39)
Lenalidomide	460923 (100)			
Human chorionic gonadotropin increased	254 (0.06)	90.75 (75.26–109.43)	90.70 (9728.69)	5.11 (4.59)
Full blood count decreased	5301 (1.15)	57.60 (55.53–59.74)	56.95 (159596.97)	4.97 (4.87)
Full blood count increased	290 (0.06)	48.42 (41.67–56.27)	48.39 (7907.35)	4.72 (4.26)
Light chain analysis increased	244 (0.05)	46.86 (39.83–55.14)	46.84 (6516.94)	4.67 (4.17)
5q minus syndrome	68 (0.01)	65.10 (46.73–90.68)	65.09 (2206.79)	4.52 (3.56)
Light chain analysis abnormal	43 (0.01)	45.59 (31.02–67.03)	45.59 (1128.67)	4.10 (2.94)
Protein total increased	320 (0.07)	22.43 (19.77–25.44)	22.41 (4939.82)	4.03 (3.62)
Laboratory test abnormal	3327 (0.72)	15.88 (15.29–16.49)	15.77 (37472.04)	3.70 (3.57)
Refractory anaemia with an excess of blasts	41 (0.01)	21.25 (14.97–30.15)	21.24 (604.59)	3.58 (2.46)
Blood stem cell harvest failure	19 (<0.01)	38.51 (21.97–67.52)	38.51 (445.35)	3.50 (1.79)
Squamous cell carcinoma of skin	470 (0.10)	11.57 (10.49–12.75)	11.56 (3881.44)	3.30 (2.98)
Blood immunoglobulin A increased	38 (0.01)	14.80 (10.42–21.02)	14.80 (402.41)	3.25 (2.12)
Pulmonary thrombosis	796 (0.17)	10.85 (10.07–11.69)	10.83 (6141.71)	3.23 (2.99)
Multiple allergies	526 (0.11)	10.82 (9.87–11.86)	10.80 (4046.14)	3.22 (2.92)
Refractory cytopenia with multilineage dysplasia	18 (<0.01)	22.97 (13.48–39.16)	22.97 (283.71)	3.22 (1.54)
Thrombosis	5506 (1.19)	10.44 (10.15–10.74)	10.33 (40394.22)	3.19 (3.09)
Malignant neoplasm of unknown primary site	43 (0.01)	12.89 (9.30–17.85)	12.88 (397.12)	3.16 (2.10)
Blood immunoglobulin G increased	72 (0.02)	11.41 (8.89–14.64)	11.41 (586.53)	3.14 (2.33)
White blood cell count decreased	6627 (1.44)	9.24 (9.01–9.48)	9.12 (42397.83)	3.03 (2.94)
Neuropathy peripheral	5857 (1.27)	9.21 (8.96–9.47)	9.11 (37384.65)	3.03 (2.94)
Pomalidomide	102810 (100)			
Full blood count decreased	1158 (1.13)	34.65 (32.60–36.83)	34.27 (33717.29)	4.92 (4.71)
Human chorionic gonadotropin increased	60 (0.06)	48.47 (36.93–63.62)	48.44 (2413.59)	4.65 (3.76)
Full blood count increased	78 (0.08)	39.02 (30.84–49.38)	38.99 (2567.19)	4.61 (3.83)
Laboratory test abnormal	908 (0.88)	16.87 (15.78–18.04)	16.73 (12752.72)	3.97 (3.75)
Pneumonia influenzal	32 (0.03)	17.33 (12.14–24.75)	17.33 (466.49)	3.49 (2.32)
Light chain analysis abnormal	14 (0.01)	46.54 (26.54–81.60)	46.54 (542.93)	3.48 (1.68)
White blood cell count decreased	1980 (1.93)	11.51 (11.00–12.04)	11.31 (17985.65)	3.45 (3.30)
Protein total increased	55 (0.05)	13.77 (10.51–18.03)	13.76 (623.31)	3.44 (2.55)
Neutrophil count decreased	608 (0.59)	10.27 (9.47–11.14)	10.22 (4899.05)	3.29 (3.02)
Blood immunoglobulin A increased	15 (0.01)	23.44 (13.87–39.61)	23.43 (299.67)	3.24 (1.56)
Neuropathy peripheral	1306 (1.27)	8.44 (7.99–8.92)	8.35 (8239.76)	3.02 (2.84)
Amyloidosis	30 (0.03)	10.84 (7.53–15.60)	10.84 (258.89)	3.01 (1.82)
Blood immunoglobulin G increased	19 (0.02)	12.17 (7.69–19.24)	12.17 (187.40)	2.93 (1.45)
Paraproteinaemia	10 (0.01)	22.64 (11.92–43.02)	22.64 (192.88)	2.90 (0.86)
Pneumonia respiratory syncytial viral	14 (0.01)	12.83 (7.52–21.89)	12.83 (146.66)	2.81 (1.10)
Multiple allergies	92 (0.09)	7.59 (6.17–9.34)	7.59 (513.68)	2.80 (2.11)
Cardiac amyloidosis	16 (0.02)	11.29 (6.85–18.59)	11.28 (144.75)	2.78 (1.18)
Malignant neoplasm of unknown primary site	11 (0.01)	13.12 (7.18–23.98)	13.12 (118.18)	2.68 (0.76)
Parainfluenzae virus infection	21 (0.02)	8.49 (5.50–13.10)	8.49 (135.06)	2.64 (1.23)
Listeriosis	16 (0.02)	8.99 (5.47–14.78)	8.99 (110.46)	2.59 (0.99)

^a^ Number of patients with adverse events.

### 3.4 Changes in the Number of IMiDs AEs reports

**FIGURE 2:**
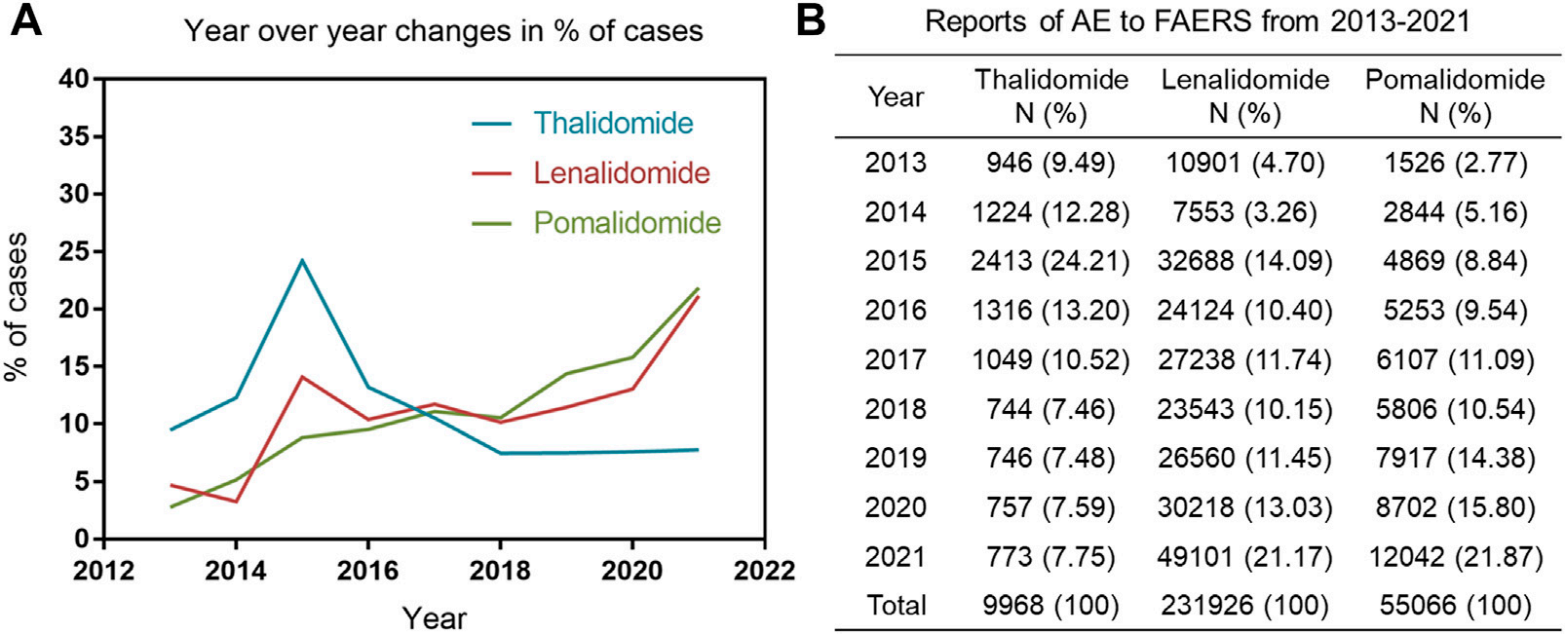
**(A)** Line graph with the percentage of AE reports of immunomodulatory drugs (IMiDs) published per year. **(B)** The number and percentage of cases reported to the food and drug administration adverse event reporting system caused by IMiDs.

Figure 2A shows line graphs with the percentage of AE reports of IMiDs (based on the number of all AEs reported for the drug over 9 years). Of those, thalidomide-related AEs reports peaked in 2015, contributing to 24.21% of all thalidomide-related AEs reported in the past 9 years, which was followed by a downward trend. However, the number of reports on lenalidomide and pomalidomide increased slowly over time. Compared with lenalidomide and pomalidomide, the thalidomide (as an old drug) related AEs reports was small in quantity in the past 9 years, with only 9968 reports (Figure 2B).

## 4 Discussion

Although IMiDs share structural similarities, their safety properties differ. However, there is a lack of published studies that evaluate post-marketing real-world AEs of IMiDs. To our knowledge, this is the first such safety study of IMiDs based on data mining of FAERS. Additionally, we focused on the differences in the associations between AEs and real-world prognosis based on the FAERS database.

Our study demonstrated that in terms of SOC, thalidomide was the only drug that showed a significant signal in “cardiac disorders,” lenalidomide showed significant signals in “gastrointestinal disorders,” and pomalidomide was strongly associated with “respiratory, thoracic, and mediastinal disorders”. The safety profiles of the IMiDs in this study were consistent with those of previous reports for individual agents. Several studies have reported that MM patients treated with thalidomide experienced arrhythmias or congenital septal defects (following its administration to pregnant women), which may be related to the interaction of the cardioprotective-related TBX5 transcriptional activator (Basson et al., 1997; Rokicka and Rokicki, 1999; Kropff et al., 2012; Khalil et al., 2017). Meanwhile, lenalidomide was more strongly associated with gastrointestinal AEs, including nausea, vomiting, diarrhea, and constipation, in a meta-analysis by Wang et al. (Wang et al., 2016). Infections were more common in patients receiving pomalidomide, and a few patients discontinued treatment because of pneumonia (Lacy et al., 2009; Leleu et al., 2013; Miguel et al., 2013; Richardson et al., 2014).

MM is associated with a high risk of VTE, and the use of IMiDs further increases this risk (Srkalovic et al., 2004; Kristinsson et al., 2010). The risk of VTE is increased by 28% and 59% when IMiDs are combined with dexamethasone (Zangari et al., 2001; Musallam et al., 2009) and chemotherapy drugs (Baz et al., 2005), respectively. Hence, the choice of drug is a major determinant of VTE risk in patients (Fotiou et al., 2016). Our results showed that pomalidomide had the lowest risk of VTE, consistent with those of previously published studies. Leclerc et al. reported that 14.7% of patients receiving lenalidomide and 13.9% of patients receiving thalidomide experienced VTE; meanwhile, only 7.4% of patients who received pomalidomide experienced VTE (Leclerc et al., 2022). The mechanisms responsible for the increased risk of VTE due to IMiD use are poorly characterized. Thalidomide has been reported to increase the levels of von Willebrand factor and factor VIII, stimulate tissue factors in monocytes, decrease thrombomodulin, and activate platelets, all of which increase the risk of VTE (Palumbo and Palladino, 2012; Abdullah et al., 2013). Lenalidomide-induced upregulation of cathepsin G, which is a platelet activator, has been suggested as a potential mechanism for the increased risk of VTE (Isozumi et al., 2013). Meanwhile, few data are available on the risk of VTE associated with pomalidomide; its incidence appears to be lower than those of thalidomide and lenalidomide, which may be related to the routine inclusion of thromboprophylaxis in the treatment regimen (Scott, 2014). We also found that lenalidomide and pomalidomide reduced the risk of peripheral neuropathy compared to thalidomide (Dalla Torre et al., 2016; Bringhen et al., 2017). Pomalidomide has good safety profiles, but AEs related to the respiratory system, especially pneumonia, cannot be ignored. Health Canada warned of an increased risk of PML in patients treated with pomalidomide, while the disproportionate analysis showed a weaker signal risk in this study with a total of 22 reports received from the FAERS database.

We observed some unexpected AEs that were not listed on the label of the drugs. The disproportionate association with human chorionic gonadotropin (hCG) was observed with all three IMiDs. A non-pregnant premenopausal woman had a positive pregnancy test after thalidomide administration (Slone et al., 2005). Additionally, Tageja et al. (2010) reported a postmenopausal woman who exhibited persistent elevations in hCG levels during lenalidomide treatment for MM. However, only a few cases of IMiDs have been reported. Hence, the risk of increased hCG levels in IMiDs remains to be demonstrated using clinical data. For thalidomide, some AEs were associated with malignancy. The ECOG E1A06 study found ten and four hematologic SPMs in MPT (melphalan, prednisone, thalidomide) and MPR (melphalan, prednisone, lenalidomide) groups, respectively (Stewart et al., 2015). The Arkansas TT2 (+/−thalidomide) trial found that thalidomide increased the risk of solid tumor SPMs and decreased the risk of hematologic malignancies (Usmani et al., 2012). Although these associations between thalidomide and SPMs are weak and unconvincing due to limited evidence, we still need to pay more attention. Multiple studies have observed an increased risk of SPMs in patients receiving lenalidomide, with the incidence of SPMs ranging from 2.6% to 8.0% (Attal et al., 2013; Holstein et al., 2015). However, this risk appears to be offset by the beneficial effects of lenalidomide on OS. A significant signal for “malignant neoplasm of unknown primary site” was found in pomalidomide, but real-world evidence is lacking. Jones et al. (2016b) reported that IMiDs may reactivate the Epstein–Barr virus (EBV), an oncogenic gamma herpes virus associated with the development and maintenance of various human malignancies, thereby enhancing the EBV lytic cycle and host immune suppression (Jha et al., 2016). Further studies are required to elucidate the molecular mechanisms underlying the association between IMiDs and SPMs. A newly suspected AE signal, Blood stem cell harvest failure, in lenalidomide has garnered our interest. In the era of novel drugs, ASCT remains the first-line treatment despite IMiDs being extremely beneficial for MM patients. However, multiple studies have shown that lenalidomide can cause myelosuppression and modify the matrix environment, thereby affecting the success rate of hematopoietic stem cell collection (Lev et al., 2006; Dupont et al., 2009; Han et al., 2012; Gao et al., 2015; Ma et al., 2016), which are consistent with our findings. Therefore, physicians should consider this risk factor when selecting various chemotherapeutic agents for patients. In summary, the discovery of novel suspected AE signals provides objective evidence for the safe and effective application of IMiDs.

Among the AE-related mortalities associated with IMiD therapy, pneumonia, sepsis, renal failure, and neutropenia ranked as the most common causes. Infectious pneumonia may be related to inherent humoral and therapy-induced immunosuppression of hematological diseases, which is an important cause of morbidity. A systematic review and meta-analysis by Chen et al. revealed that RRMM patients receiving pomalidomide had the highest rate of severe infections in randomized controlled trials and observational studies (Chen et al., 2018), which are consistent with our findings. Sepsis is triggered by infection, and neutropenia increases the risk of infection. Lenalidomide combined with high-dose dexamethasone resulted in grade 3–4 infections in 10%–22% of patients with MM (Dimopoulos et al., 2007; Weber et al., 2007). Meanwhile, grade 3–4 infections occurred in 7%–14% of MM patients treated with thalidomide and glucocorticoid (Palumbo et al., 2006; Facon et al., 2007; Rajkumar et al., 2008). Furthermore, a severe infection rate of 23% was observed among patients with MM undergoing pomalidomide-based regimens (Chen et al., 2018). Moreover, IMiDs also exhibit different pharmacological profiles. The metabolic pathway of lenalidomide is mainly related to renal function (Jelinek et al., 2016). Similarly, the toxicity of thalidomide on renal function is not negligible (Seldin et al., 2003). In contrast, pomalidomide shows promising efficacy and favorable toxicity profiles in patients with renal insufficiency (Jelinek et al., 2016). Renal insufficiency is a typical clinical finding in patients with MM. Therefore, assessment of renal function is recommended before selecting therapeutic regimens to avoid aggravating renal failure and accelerating patient death.

During our study period, the thalidomide-related AEs reports was small in quantity, which may be due to the increased use of lenalidomide and pomalidomide. Additionally, reports of thalidomide causing congenital deformities in neonates have resulted in its decreased use. Although it was approved for treatment of MM, its use has not been widespread compared to the other IMiDs (Millen, 1962). Furthermore, we did not observe the Weber effect in AE reports of IMiDs, and the reason for this is likely multifactorial. First, the pharmaceutical industry and the general public have gradually increased their awareness of drug safety, and AE prevention has received greater attention (Hoffman et al., 2014). Second, institutions engaged in risk evaluation and mitigation strategies, more stringent regulatory authorities, and the convenience brought by the internet have promoted the reporting of AEs (United States Food and Drug Administration (USFDA), 2017; United States Food and Drug Administration (USFDA), 2022; Ilic, 2010; Hart et al., 2004).

Data mining of FAERS can effectively compensate for the shortcomings of clinical trials, such as a small sample size, narrow coverage, and short observation time; however, there are still some limitations regarding this method. First, most reports in FAERS are from the United States, and the results of this study may not be generalizable due to variations in drug usage and ethnicity among different countries (Sakaeda et al., 2013). Second, since the FAERS database is a spontaneous reporting system, some problems inevitably occur, such as underreporting and incomplete or inaccurate reporting. Therefore, bias in the results is expected (Pariente et al., 2007). Finally, the FDA has no requirement for demonstrating the causal involvement of AEs and drugs before reporting. Thus, the risk signals obtained by disproportionality analysis can only indicate statistical significance rather than biological significance (FDA Adverse Events Reporting System (FAERS) Public Dashboard, 2022). Overall, our results do not represent the inevitable causal relationship between a drug and AE. Nonetheless, the FAERS database remains a unique and important tool for post-marketing safety surveillance of approved drugs.

## 5 Conclusion

We reviewed the safety profiles of thalidomide, lenalidomide, and pomalidomide based on AEs submitted to the FAERS database from 2013Q1 to 2021Q4. According to 296,960 reports, AEs with IMiDs occurred in multiple organs and tissues, including the cardiac, vascular, respiratory, and integumentary systems. IMiDs have different safety profiles that may cause serious AEs, resulting in treatment discontinuation or patient mortality. Clinicians should be aware of these differences and adjust treatment regimens for different patients to improve patient compliance and reduce the risk of AEs. Although several post-marketing safety signals that were off label were found, prospective clinical trials are necessary to confirm these findings.

## Data Availability

Publicly available datasets were analyzed in this study. This data can be found here: https://fis.fda.gov/extensions/FPD-QDE-FAERS/FPD-QDE-FAERS.html.
