# Supplementary material for: Post-marketing safety of immunomodulatory drugs in multiple myeloma: A pharmacovigilance investigation based on the FDA adverse event reporting system
Source: Front Pharmacol. 2022 Dec 1;13:989032. doi: 10.3389/fphar.2022.989032 (PMC9751748; doi:10.3389/fphar.2022.989032)
Supplement: Supplementary file 1 [file Table1.docx]

**Table S1** Two-by-two contingency table for disproportionality analyses.

|  | **Reports with the target AEs** | **All other AEs** | **Total** |
| --- | --- | --- | --- |
| Target drug | a | b | a+b |
| All other drugs | c | d | c+d |
| Total | a+c | b+d | a+b+c+d |
