# Supplementary material for: Post-marketing safety of immunomodulatory drugs in multiple myeloma: A pharmacovigilance investigation based on the FDA adverse event reporting system
Source: Front Pharmacol. 2022 Dec 1;13:989032. doi: 10.3389/fphar.2022.989032 (PMC9751748; doi:10.3389/fphar.2022.989032)
Supplement: Supplementary file 2 [file Table2.docx]

**Table S2** Summary of major algorithms used for signal detection.

| **Algorithms** | **Calculation formula** | **Criteria** |
| --- | --- | --- |
| ROR | ROR=(a/b)/(c/d) | a ≥ 3, 95%CI > 1 |
| PRR | PRR = (a/(a + c))/(b/(b + d)) | a ≥ 3, PRR ≥ 2, χ^2^ ≥ 4 |
| BCPNN | IC = log_2_^a (a + b + c + d)/((a + c) (a + b))^ | IC-2SD > 0 |
