# Supplementary material for: Post-marketing safety of immunomodulatory drugs in multiple myeloma: A pharmacovigilance investigation based on the FDA adverse event reporting system
Source: Front Pharmacol. 2022 Dec 1;13:989032. doi: 10.3389/fphar.2022.989032 (PMC9751748; doi:10.3389/fphar.2022.989032)
Supplement: Supplementary file 3 [file Table3.docx]

**Table S3** Distribution of drug-reaction pairs attributed to immunomodulatory drugs (IMiDs) according to relevant system organ class (SOC).

| **SOC** | **Thalidomide**  **N^a^ (%)** | **Lenalidomide**  **N^a^ (%)** | **Pomalidomide**  **N^a^ (%)** |
| --- | --- | --- | --- |
| Infections and infestations | 210 (10.10) | 654 (11.53) | 395 (13.45) |
| Neoplasms benign, malignant and unspecified (incl cysts and polyps) | 207 (9.95) | 648 (11.43) | 216 (7.35) |
| Investigations | 177 (8.51) | 603 (10.63) | 306 (10.42) |
| Nervous system disorders | 177 (8.51) | 359 (6.33) | 207 (7.05) |
| Gastrointestinal disorders | 155 (7.45) | 434 (7.65) | 224 (7.63) |
| General disorders and administration site conditions | 122 (5.87) | 254 (4.48) | 158 (5.38) |
| Skin and subcutaneous tissue disorders | 115 (5.53) | 227 (4.00) | 133 (4.53) |
| Injury, poisoning and procedural complications | 114 (5.48) | 368 (6.49) | 190 (6.47) |
| Respiratory, thoracic and mediastinal disorders | 95 (4.57) | 223 (3.93) | 145 (4.94) |
| Cardiac disorders | 86 (4.13) | 141 (2.49) | 89 (3.03) |
| Psychiatric disorders | 83 (3.99) | 179 (3.16) | 101 (3.44) |
| Musculoskeletal and connective tissue disorders | 81 (3.89) | 216 (3.81) | 124 (4.22) |
| Vascular disorders | 63 (3.03) | 140 (2.47) | 78 (2.66) |
| Metabolism and nutrition disorders | 57 (2.74) | 124 (2.19) | 82 (2.79) |
| Renal and urinary disorders | 52 (2.50) | 149 (2.63) | 80 (2.72) |
| Eye disorders | 51 (2.45) | 183 (3.23) | 87 (2.96) |
| Blood and lymphatic system disorders | 48 (2.31) | 115 (2.03) | 52 (1.77) |
| Hepatobiliary disorders | 37 (1.78) | 83 (1.46) | 40 (1.36) |
| Immune system disorders | 30 (1.44) | 71 (1.25) | 45 (1.53) |
| Reproductive system and breast disorders | 29 (1.39) | 153 (2.70) | 60 (2.04) |
| Endocrine disorders | 18 (0.87) | 43 (0.76) | 16 (0.54) |
| Social circumstances | 17 (0.82) | 47 (0.83) | 19 (0.65) |
| Ear and labyrinth disorders | 16 (0.77) | 45 (0.79) | 28 (0.95) |
| Surgical and medical procedures | 13 (0.63) | 112 (1.98) | 34 (1.16) |
| Product issues | 12 (0.58) | 34 (0.60) | 16 (0.54) |
| Congenital, familial and genetic disorders | 11 (0.53) | 56 (0.99) | 11 (0.37) |
| Pregnancy, puerperium and perinatal conditions | 4 (0.19) | 9 (0.16) | 1 (0.03) |
| Total | 2080 (100) | 5670 (100) | 2937 (100) |

^a^ Number of patients with adverse events.
