# Supplementary material for: Post-marketing safety of immunomodulatory drugs in multiple myeloma: A pharmacovigilance investigation based on the FDA adverse event reporting system
Source: Front Pharmacol. 2022 Dec 1;13:989032. doi: 10.3389/fphar.2022.989032 (PMC9751748; doi:10.3389/fphar.2022.989032)
Supplement: Supplementary file 4 [file Table4.docx]

**Table S4** Top 20 preferred terms (PT) associated with immunomodulatory drugs (IMiDs) for reported numbers.

| **PT** | **Na (%)** | **ROR (95% CI)** | **PRR (χ2)** | **IC (IC-2SD)** |
| --- | --- | --- | --- | --- |
| **Thalidomide** | 21045 (100) |  |  |  |
| Neuropathy peripheral | 544 (2.58%) | 17.19 (15.78-18.72) | 16.77 (7991.69) | 4.01 (3.73) |
| Pneumonia | 362 (1.72) | 3.25 (2.93-3.61) | 3.21 (553.56) | 1.67 (1.33) |
| Unevaluable event | 306 (1.45) | 10.52 (9.39-11.78) | 10.38 (2580.15) | 3.32 (2.95) |
| Adverse drug reaction | 288 (1.37) | 9.65 (8.58-10.84) | 9.53 (2187.81) | 3.20 (2.82) |
| Constipation | 276 (1.31) | 3.82 (3.39-4.31) | 3.79 (566.55) | 1.90 (1.51) |
| Anaemia | 177 (0.84) | 2.67 (2.30-3.09) | 2.65 (182.74) | 1.39 (0.90) |
| Deep vein thrombosis | 171 (0.81) | 7.72 (6.64-8.98) | 7.67 (987.52) | 2.88 (2.38) |
| Thrombosis | 167 (0.79) | 6.11 (5.25-7.12) | 6.07 (705.86) | 2.55 (2.05) |
| Hypoaesthesia | 167 (0.79) | 3.23 (2.78-3.77) | 3.22 (255.28) | 1.66 (1.16) |
| Peripheral swelling | 142 (0.67) | 2.25 (1.91-2.65) | 2.24 (97.48) | 1.15 (0.60) |
| Renal failure | 141 (0.67) | 2.86 (2.43-3.38) | 2.85 (169.61) | 1.49 (0.94) |
| Pulmonary embolism | 134 (0.64) | 4.32 (3.65-5.12) | 4.30 (338.99) | 2.07 (1.50) |
| Full blood count decreased | 131 (0.62) | 17.41 (14.64-20.69) | 17.30 (1990.61) | 3.93 (3.36) |
| Drug intolerance | 131 (0.62) | 3.71 (3.12-4.40) | 3.69 (256.72) | 1.85 (1.28) |
| Sepsis | 124 (0.59) | 3.28 (2.74-3.91) | 3.26 (194.43) | 1.68 (1.09) |
| Paraesthesia | 119 (0.57) | 2.23 (1.87-2.68) | 2.23 (80.52) | 1.14 (0.54) |
| Infection | 117 (0.56) | 2.40 (2.00-2.88) | 2.39 (95.13) | 1.24 (0.64) |
| Platelet count decreased | 112 (0.53) | 3.18 (2.64-3.82) | 3.16 (165.72) | 1.63 (1.02) |
| Neutropenia | 112 (0.53) | 2.74 (2.27-3.30) | 2.73 (122.75) | 1.42 (0.81) |
| Haemoglobin decreased | 111 (0.53) | 3.24 (2.69-3.90) | 3.23 (170.50) | 1.24 (1.02) |
| **Lenalidomide** | 460923 (100) |  |  |  |
| Diarrhea | 15527 (3.37) | 3.33 (3.27-3.38) | 3.25 (23322.73) | 1.65 (1.60) |
| Fatigue | 13794 (2.99) | 2.35 (2.31-2.39) | 2.31 (10085.35) | 1.18 (1.13) |
| Pneumonia | 10916 (2.37) | 4.74 (4.64-4.83) | 4.65 (29434.25) | 2.14 (2.08) |
| Rash | 9150 (1.99) | 2.95 (2.89-3.02) | 2.91 (11113.55) | 1.50 (1.43) |
| White blood cell count decreased | 6627 (1.44) | 9.24 (9.01-9.48) | 9.12 (42397.83) | 3.03 (2.94) |
| Neuropathy peripheral | 5857 (1.27) | 9.21 (8.96-9.47) | 9.11 (37384.65) | 3.03 (2.94) |
| Thrombosis | 5506 (1.19) | 10.44 (10.15-10.74) | 10.33 (40394.22) | 3.19 (3.09) |
| Constipation | 5444 (1.18) | 3.56 (3.46-3.66) | 3.53 (9409.26) | 1.77 (1.68) |
| Full blood count decreased | 5301 (1.15) | 57.60 (55.53-59.74) | 56.95 (159596.97) | 4.97 (4.87) |
| Platelet count decreased | 5295 (1.15) | 7.52 (7.31-7.74) | 7.45 (26727.60) | 2.77 (2.67) |
| Muscle spasms | 4205 (0.91) | 2.97 (2.88-3.06) | 2.95 (5218.27) | 1.52 (1.42) |
| Peripheral swelling | 3751 (0.81) | 2.78 (2.69-2.87) | 2.77 (4078.69) | 1.43 (1.32) |
| Adverse drug reaction | 3533 (0.77) | 5.70 (5.50-5.90) | 5.66 (12546.91) | 2.41 (2.29) |
| Neutropenia | 3500 (0.76) | 4.08 (3.95-4.23) | 4.06 (7638.53) | 1.96 (1.85) |
| Unevaluable event | 3486 (0.76) | 5.77 (5.57-5.97) | 5.73 (12583.15) | 2.42 (2.31) |
| Anaemia | 3333 (0.72) | 2.33 (2.25-2.41) | 2.32 (2436.27) | 1.19 (1.07) |
| Laboratory test abnormal | 3327 (0.72) | 15.88 (15.29-16.49) | 15.77 (37472.04) | 3.70 (3.57) |
| Haemoglobin decreased | 3027 (0.66) | 4.22 (4.06-4.37) | 4.20 (6955.40) | 2.00 (1.88) |
| Deep vein thrombosis | 2880 (0.62) | 6.35 (6.11-6.60) | 6.32 (11819.00) | 2.55 (2.42) |
| Dehydration | 2687 (0.58) | 2.84 (2.73-2.95) | 2.83 (3059.61) | 1.46 (1.33) |
| **Pomalidomide** | 102810 (100) |  |  |  |
| Pneumonia | 3683 (3.58) | 7.02 (6.79-7.26) | 6.81 (17946.75) | 2.74 (2.63) |
| Fatigue | 3299 (3.21) | 2.49 (2.41-2.58) | 2.45 (2835.70) | 1.28 (1.17) |
| White blood cell count decreased | 1980 (1.93) | 11.51 (11.00-12.04) | 11.31 (17985.65) | 3.45 (3.30) |
| Asthenia | 1328 (1.29) | 2.13 (2.02-2.25) | 2.12 (784.17) | 1.08 (0.90) |
| Neuropathy peripheral | 1306 (1.27) | 8.44 (7.99-8.92) | 8.35 (8239.76) | 3.02 (2.84) |
| Constipation | 1176 (1.14) | 3.35 (3.16-3.55) | 3.32 (1894.14) | 1.72 (1.53) |
| Full blood count decreased | 1158 (1.13) | 34.65 (32.60-36.83) | 34.27 (33717.29) | 4.92 (4.71) |
| Neutropenia | 1058 (1.03) | 5.39 (5.07-5.73) | 5.34 (3681.11) | 2.39 (2.19) |
| Platelet count decreased | 971 (0.94) | 5.74 (5.38-5.73) | 5.69 (3693.96) | 2.48 (2.27) |
| Laboratory test abnormal | 908 (0.88) | 16.87 (15.78-18.04) | 16.73 (12752.72) | 3.97 (3.75) |
| Nasopharyngitis | 903 (0.88) | 2.86 (2.68-3.06) | 2.85 (1074.65) | 1.50 (1.28) |
| Peripheral swelling | 887 (0.86) | 2.89 (2.71-3.09 | 2.88 (1079.82) | 1.51 (1.29) |
| Back pain | 859 (0.84) | 2.13 (1.99-2.28) | 2.12 (507.07) | 1.08 (0.85) |
| Adverse drug reaction | 814 (0.79) | 5.60 (5.22-6.00) | 5.56 (2996.60) | 2.45 (2.22) |
| Thrombosis | 810 (0.79) | 6.15 (5.73-6.59) | 6.11 (3398.08) | 2.58 (2.35) |
| Unevaluable event | 764 (0.74) | 5.38 (5.01-5.78) | 5.35 (2657.71) | 2.39 (2.15) |
| Influenza | 763 (0.74) | 4.02 (3.74-4.32) | 4.00 (1696.62) | 1.98 (1.74) |
| Infection | 763 (0.74) | 3.23 (3.01-3.47) | 3.22 (1156.35) | 1.67 (1.43) |
| Muscle spasms | 728 (0.71) | 2.25 (2.09-2.42) | 2.24 (496.13) | 1.15 (0.91) |
| Anaemia | 708 (0.69) | 2.19 (2.03-2.36) | 2.18 (449.98) | 1.12 (0.87) |

^a^ Number of patients with adverse events.
